# Investigation of trends, hot spots and effective therapies for pregnancy outcomes in polycystic ovary syndrome: a bibliometric analysis

**DOI:** 10.3389/fmed.2026.1756551

**Published:** 2026-05-07

**Authors:** Zhenyu Shi, Fengjuan Li, Tianhang Gao, Hui Chang

**Affiliations:** 1 The First Clinical Medical College, Heilongjiang University of Chinese Medicine, Harbin, China; 2 Department of Obstetrics and Gynecology, First Affiliated Hospital of Heilongjiang University of Chinese Medicine, Harbin, China

**Keywords:** bibliometrics, CiteSpace, polycystic ovary syndrome, pregnancy outcome, VOSviewer

## Abstract

**Background:**

Polycystic Ovary Syndrome (PCOS) is among the most prevalent endocrine disorders in women of reproductive age and may significantly impact pregnancy outcomes. However, no comprehensive bibliometric analysis has synthesized research findings in this field.

**Objective:**

This study aims to identify trends and key research areas in PCOS and pregnancy outcomes and to summarize effective treatment strategies for PCOS-related infertility.

**Method:**

Literature was retrieved and collated from the Web of Science Core Collection (WOSCC) and PubMed databases up to 10 May 2025. Bibliometric analysis was conducted using VOSviewer, CiteSpace, and R software.

**Result:**

A total of 410 WOSCC publications and 45 PubMed clinical trials were included. China (*n* = 192, 46.8%) and the United States (*n* = 52, 12.7%) were the leading contributors. Shanghai Jiao Tong University had the greatest output, while the University of Adelaide was the most cited institution. Vanky, Eszter was the most prolific author, and Legro, Richard S. was the most cited. Frontiers in Endocrinology was the leading core journal. Insulin resistance and metformin were identified as current research hotspots, while neonatal outcomes, metabolic syndrome, and ovarian hyperstimulation syndrome represent emerging frontiers.

**Conclusion:**

This first bibliometric analysis of PCOS and pregnancy outcomes provides a comprehensive overview of the research landscape. Future research should focus on insulin resistance mechanisms, metabolic syndrome management, and the optimization of assisted reproductive technologies including frozen embryo transfer, letrozole combination therapies, and traditional Chinese medicine approaches.

## 1 Introduction

Polycystic Ovary Syndrome (PCOS) is a common hormonal disorder that affects about 10%−13% of women worldwide during their reproductive years (1). Characterized by hyperandrogenemia (HA), insulin resistance (IR), and obesity, PCOS can significantly impact pregnancy outcomes and long-term health (2). Infertility remains the primary concern for most PCOS patients, with many opting for fertility treatments or even requiring assisted reproductive technologies (ART) to conceive (3). Individuals who become pregnant are at increased risk for gestational complications, including gestational diabetes mellitus (GDM), preeclampsia, and placental abruption (4). These conditions may result in adverse pregnancy outcomes such as preterm birth, fetal growth restriction, and low birth weight. These complications not only strain family wellbeing but also pose challenges to social stability. Therefore, advancing research in PCOS and infertility management is crucial for public health and societal welfare.

Pritchard first introduced bibliometric analysis in 1969 (5), which has since been widely used to evaluate research trends via mathematical and statistical methods for assessing the academic impact and characteristics of scientific publications (6). This method enables quantitative analysis of literature, identification of research hotspots and trends, and prediction of research frontiers (7). VOSviewer software, developed by van Eck and Waltman (8), can visually display the relationship between different terms. CiteSpace is a visual analysis tool created by Chen Chaomei. It combines cluster analysis with social network analysis (9). The Biblioshiny online platform is an interactive application developed using the R programming language (R Core Team, R Foundation for Statistical Computing, Vienna, Austria; https://www.Rproject.org) and the Bibliometrix package [Massimo Aria and Corrado Cuccurullo, University of Naples Federico II (University of Naples Parthenope), Naples, Italy; https://www.bibliometrix.org]. It is specifically designed to facilitate integrated scientific mapping, analysis, and the visualization of research results (10). All 3 software are indispensable and important tools in bibliometric analysis. Although bibliometric methods are widely applied in the study of PCOS, analyses specifically addressing pregnancy outcomes remain absent. We aim to use VOSviewer, CiteSpace, and Biblioshiny to identify emerging research directions and key topics related to PCOS and pregnancy outcomes.

To our knowledge, this represents the first comprehensive literature review systematically evaluating and synthesizing research findings in the field of PCOS and pregnancy outcomes. The study collected scientific publications on PCOS and pregnancy outcomes from the Web of Science Core Collection (WOSCC) database and PubMed database over multiple years, followed by a detailed bibliometric analysis. This research aims to systematically organize existing scientific literature, helping researchers quickly identify the most active hotspots and future trends in the PCOS-pregnancy outcomes field. It also summarizes current effective strategies or emerging therapies for treating PCOS-related infertility, providing valuable guidance for both scholars and clinicians.

## 2 Method

### 2.1 Data retrieval

**Figure 1:**
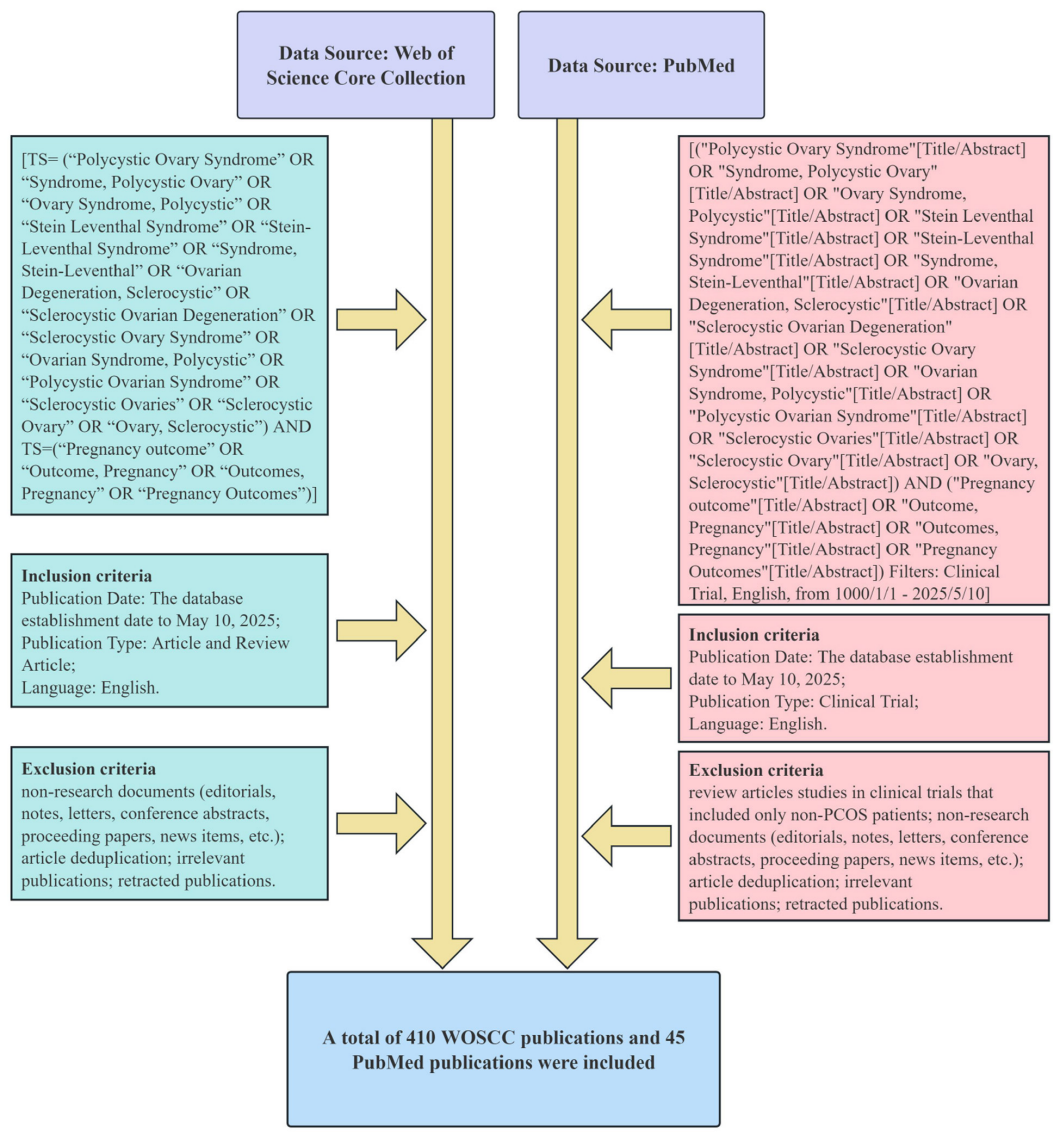
The process of screening the literature.

In the WOSCC database, the search strategy employed Topic Search (TS) combining PCOS-related terms (including “Polycystic Ovary Syndrome,” “Syndrome, Polycystic Ovary,” “Stein Leventhal Syndrome,” “Ovarian Degeneration, Sclerocystic,” and related variants) connected by the Boolean operator OR, combined with pregnancy outcome-related terms (“Pregnancy outcome,” “Outcome, Pregnancy,” “Outcomes, Pregnancy,” “Pregnancy Outcomes”) using the AND operator. In the PubMed database, an analogous strategy was applied using Title/Abstract field tags with the same Boolean logic. The complete search strings for both databases are presented in Figure 1 and Supplementary Table 1. Supplementary Table 1, including the complete search strategies for both databases, are available as Supplementary Table 1 and complement the main text by providing full reproducibility details. This approach achieves complementary strengths and mutual corroboration of findings.

### 2.2 Inclusion and exclusion criteria

Data has been collected from 2 databases covering the period from their establishment until 10 May 2025. The WOSCC database exclusively includes English-language literature classified as either “article” or “review article”; the PubMed database exclusively includes English-language articles classified as “clinical trial.” Both databases exclude publications such as editorials, notes, letters, conference abstracts, proceedings papers, news items, and retracted publications. Furthermore, PubMed excluded review articles and studies in clinical trials that included only non-PCOS patients. We manually reviewed each title and abstract, then removed irrelevant literature and deleted duplicate items to ensure all articles were unique. The literature underwent independent screening. Discrepancies were adjudicated by a third reviewer.

### 2.3 Data analysis

Using VOSviewer (Leiden University, Netherlands, version 1.6.20) and CiteSpace (Chaomei Chen, Drexel University, Philadelphia, PA, USA; version 6.4.R2) alongside the Bibliometrix package in R software, the included literature was presented visually. This provided clear visualizations and highlighted research hotspots and trends. Import all valid data into VOSviewer (version 1.6.20) for visualization of countries, institutions, authors, journals, co-citations, and keywords, and import CiteSpace (version 6.4.R2) for burst detection of keywords and co-citations. The study also used a variety of tools, including EndNote, WPS Office, and Microsoft Excel 2021, for data collection and plotting. Specific data transformations were applied during the visual analysis process to maintain consistency. First, keywords representing the same concept but differing in spelling or phrasing were merged (e.g., “polycystic ovary syndrome” and “PCOS”; “*in vitro* fertilization” and “IVF”). Second, publication data from Hong Kong and Taiwan were merged into China's data, following the geopolitical classification commonly adopted in bibliometric studies using the WOSCC database, where institutional affiliations are linked to their respective countries or regions. We acknowledge that this merging approach may slightly inflate the Chinese mainland's publication count and could obscure regional differences in research focus, methodology, and quality. Country-level data should be interpreted within the appropriate contextual framework.

## 3 Result

### 3.1 Trends in annual publications from the WOSCC database

**Figure 2:**
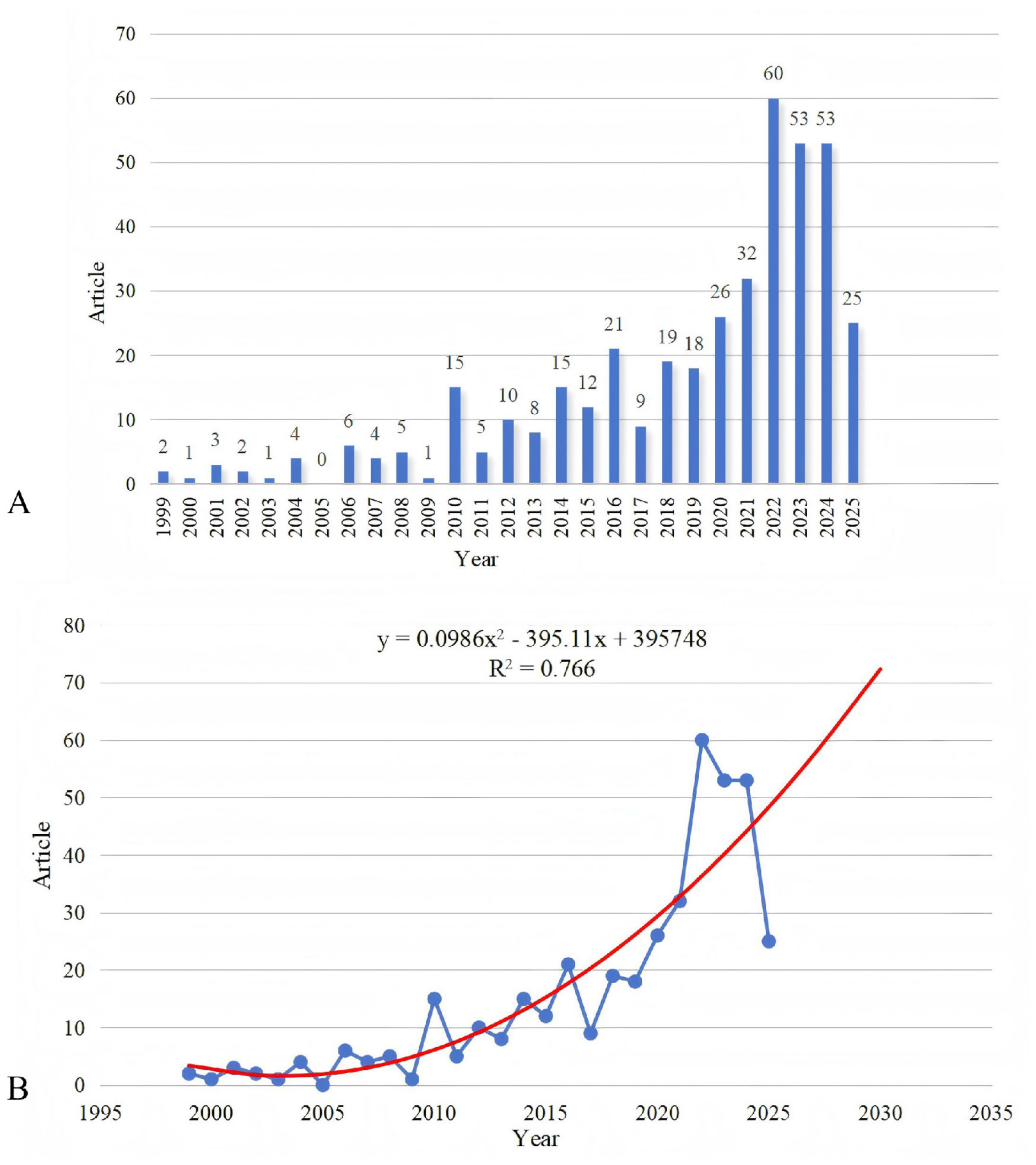
**(A)** Annual number of publications. **(B)** Publishing trends in the field are depicted. The blue line indicates actual publication counts, while the red line shows predicted counts.

Between the inception of the WOSCC database and May 10, 2025, a total of 2,023 authors from 667 institutions across 58 countries or regions published 410 papers in 152 journals, referencing 11,190 documents from 2,150 journals. Of the total, 345 were classified as original research articles (84.15%), while 65 were identified as review articles (15.85%). As shown in Figure 2A, publications were relatively scarce between 1999 and 2009. A notable increase occurred in 2010, followed by fluctuating numbers from 2011 to 2018 without significant growth. The trend only became consistent with annual increases from 2019 to 2022. However, there was a decline in 2023. As of May 10, 2025, the total number of publications reached 25, though an overall upward trajectory continues. This trend suggests that research on PCOS and pregnancy outcomes was in its infancy in the early years, and the research on PCOS and pregnancy outcomes was a hot topic in academia between 2019 and 2022, with a decline in popularity after 2023. Therefore, we built a polynomial regression model to predict the number of annual publications (*R*^2^ = 0.766), and predicted that the number of publications would exceed 70 by 2030 (Figure 2B).

### 3.2 Country

**Figure 3:**
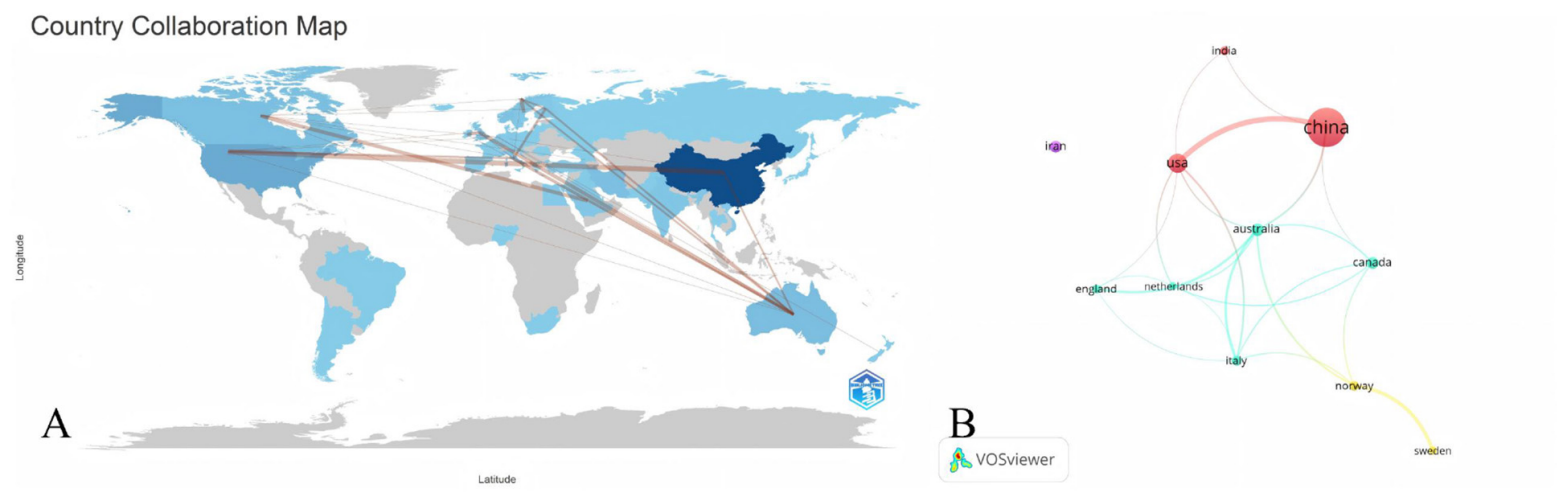
**(A)** A collaboration map among countries, where the width of the red lines indicates the degree of cooperation between countries. Shades of blue indicate the number of articles each country has published; deeper colors correspond to a larger publication volume. **(B)** A visual network of the top 11 countries/regions in terms of publication volume. The lines represent interconnections between countries, with thicker lines indicating stronger connections. Colors indicate clusters; nodes with identical colors are assigned to the same cluster.

Research on PCOS and pregnancy outcomes has received significant contributions from 58 countries worldwide. Table 1 lists the 11 countries or regions that have published the most in this domain. Among them, China has the most publications, with 192 articles (46.8%). These publications have accumulated 2,406 citations in total, with an average of 13 citations per paper. The United States ranked second, contributing 52 articles (12.7%) that collectively received 2,796 citations, resulting in an average of 54 citations per article. Australia claimed third place with 24 articles, making up 5.9% of the total, while Iran and Canada closely trailed behind with 22 (5.4%) and 21 (5.1%) articles, respectively. When examining the top 11 countries in terms of publication quantity rankings, we found that although the Netherlands ranks relatively low, its average citation count is the highest (107 times), indicating that its research quality far surpasses all other countries. While China leads in overall ranking, its average citation count lags behind the other top 11 countries, hinting that its research may not match their quality. The visualization of cooperative networks among the top 11 countries in terms of publication volume formed 4 stable clusters. China demonstrated cooperative relationships with the United States, Australia, India, and Canada, particularly showing close cooperation with the United States. Australia, Netherlands, and Italy cooperated most with other countries, while Iran did not cooperate with any country (Figures 3A, 3B).

**Table 1:** Countries/regions with the top 11 number of publications.

Rank	Country	Documents (*n*)	Percentage (*n*/410)	Citations	Average citations
1	China	192	46.8	2,406	13
2	USA	52	12.7	2,796	54
3	Australia	24	5.9	1,391	58
4	Iran	22	5.4	198	9
5	Canada	21	5.1	1,246	59
6	Norway	16	3.9	793	50
7	Italy	15	3.7	1,155	77
8	England	12	2.9	503	42
9	India	12	2.9	137	11
10	Netherlands	11	2.7	1,174	107
11	Sweden	11	2.7	482	44

### 3.3 Institution

**Figure 4:**
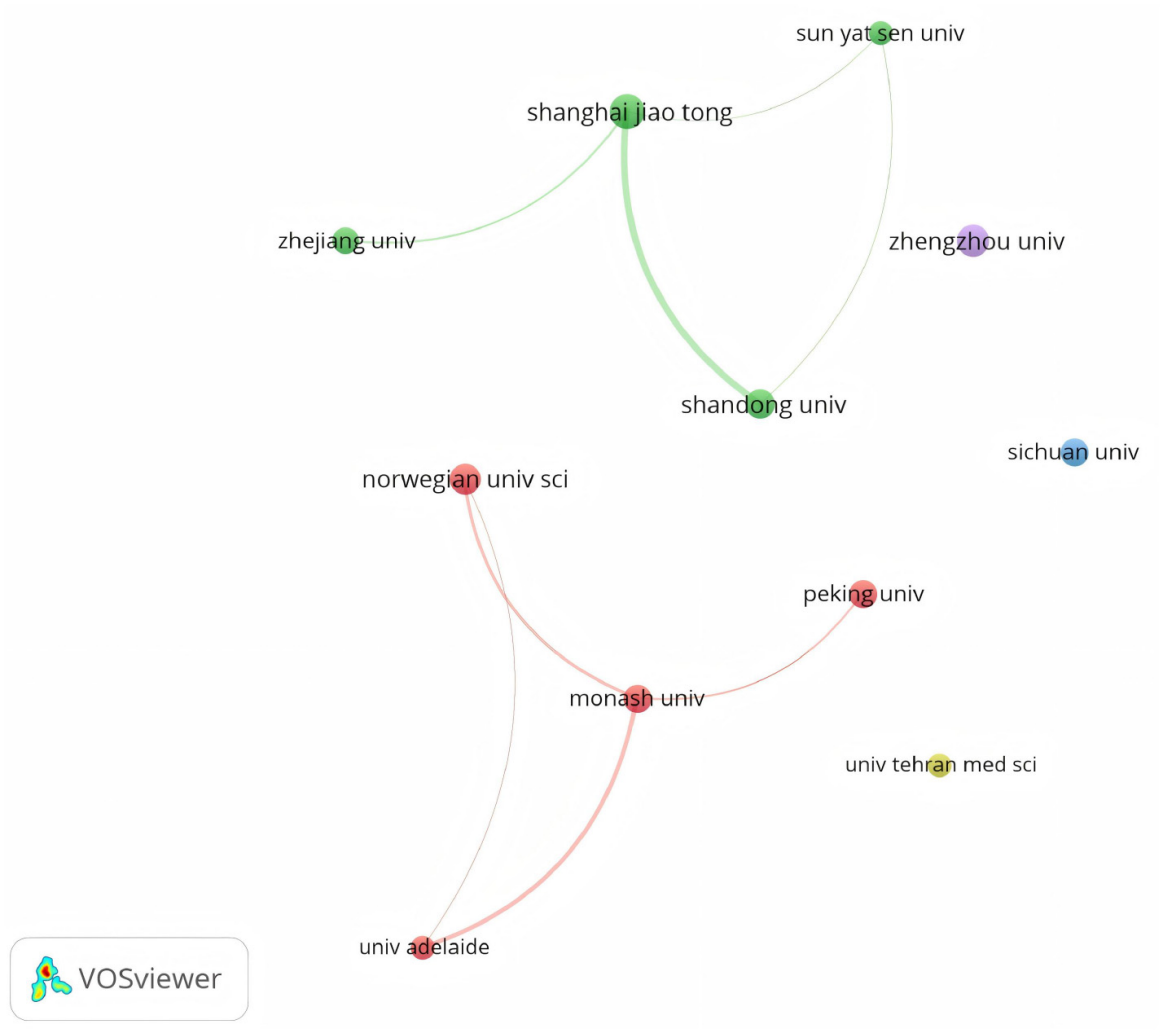
A visual network of the top 11 institutions by publication volume. Nodes represent institutions, with larger nodes indicating higher paper output. Lines demonstrate institutional connections, where thicker lines signify stronger relationships. Colors indicate clusters; nodes with identical colors are assigned to the same cluster.

A total of 667 institutions contributed to the literature on PCOS and pregnancy outcomes. Table 2 highlights the top 10 institutions by publication count and citations. Leading the pack, Shanghai Jiao Tong University claimed the top spot with 20 publications. Close behind were Zhengzhou University with 17 papers, the Norwegian University of Science and Technology with 16, Shandong University with 15, and Monash University with 13. Most of these institutions are located in China. In terms of citations, the University of Adelaide topped the list with 949 citations, followed by the Norwegian University of Science and Technology with 793 citations and The Pennsylvania State University with 784 citations. Network visualization analysis of the top 11 institutions (based on publication volume) indicates the formation of 5 clusters, which are presented in Figure 4. Among them, Shanghai Jiao Tong University and Shandong University showed close collaboration, while Zhengzhou University, Sichuan University, and Tehran University of Medical Sciences had no partnerships with any institutions.

**Table 2:** The top 11 publishers by number of publications.

Rank	Institutions	Country	Documents	Citations	Average citations
1	Shanghai Jiao Tong University	China	20	339	17
2	Zhengzhou University	China	17	189	11
3	Norwegian University of Science and Technology	Norway	16	793	50
4	Shandong University	China	15	176	12
5	Monash University	Australia	13	506	39
6	Peking University	China	13	151	12
7	Sichuan University	China	13	82	6
8	Zhejiang University	China	12	121	10
9	Sun Yat Sen University	China	10	112	11
10	The University of Adelaide	Australia	9	949	105
11	Tehran University of Medical Sciences	Iran	9	134	15

### 3.4 Author

A total of 2,023 authors contributed to these articles. Analyzing literature authorship helps identify key scholars and leading research groups in the field. Price (88) found that, for a given topic, half of the publications are written by a small group of highly productive researchers. The size of this group is about the square root of the total number of authors, as shown below:


$$\begin{array}{l} \overset{I}{\underset{m+1}{\sum }}n\left(x\right)=\sqrt{N} \end{array}$$

In this context, 3 key variables are defined as follows: *n*(x) is the count of authors who have published x papers; I = nmax is the maximum number of papers by the field's most prolific author (nmax = 15, calculated using VOSviewer); and *N* is the total number of authors, while m is the number of papers published by core authors. Based on Price's Law, the minimum number of papers that core authors in this field must publish is m = 0.749 ($\sqrt{n\max}$) ≈ 2.90. Therefore, authors with 3 or more publications were designated as core contributors in this field. There were a total of 103 core authors, and a total of 166 papers were published, accounting for 40% of the total number of papers, basically reaching the half standard (50%) proposed by Price. The above values were also basically consistent with the calculation formula of Price's Law. These findings indicate the establishment of a stable and collaborative research community focused on pregnancy outcomes in PCOS. Table 3 lists the 10 leading authors in this field.

**Table 3:** The top 10 authors with the highest number of publications.

Rank	Author	Documents	Citations	Average citations
1	Vanky, Eszter	15	792	53
2	Li, Rong	11	112	10
3	Legro, Richard S.	9	884	98
4	Qiao, Jie	9	114	13
5	Teede, Helena J.	9	372	41
6	Zhang, Heping	8	72	9
7	Dahan, Michael H.	8	699	87
8	Daghlaf, Haitham	7	85	12
9	Carlsen, Sven M.	7	667	95
10	Glueck, Charles J.	7	667	95

**Figure 5:**
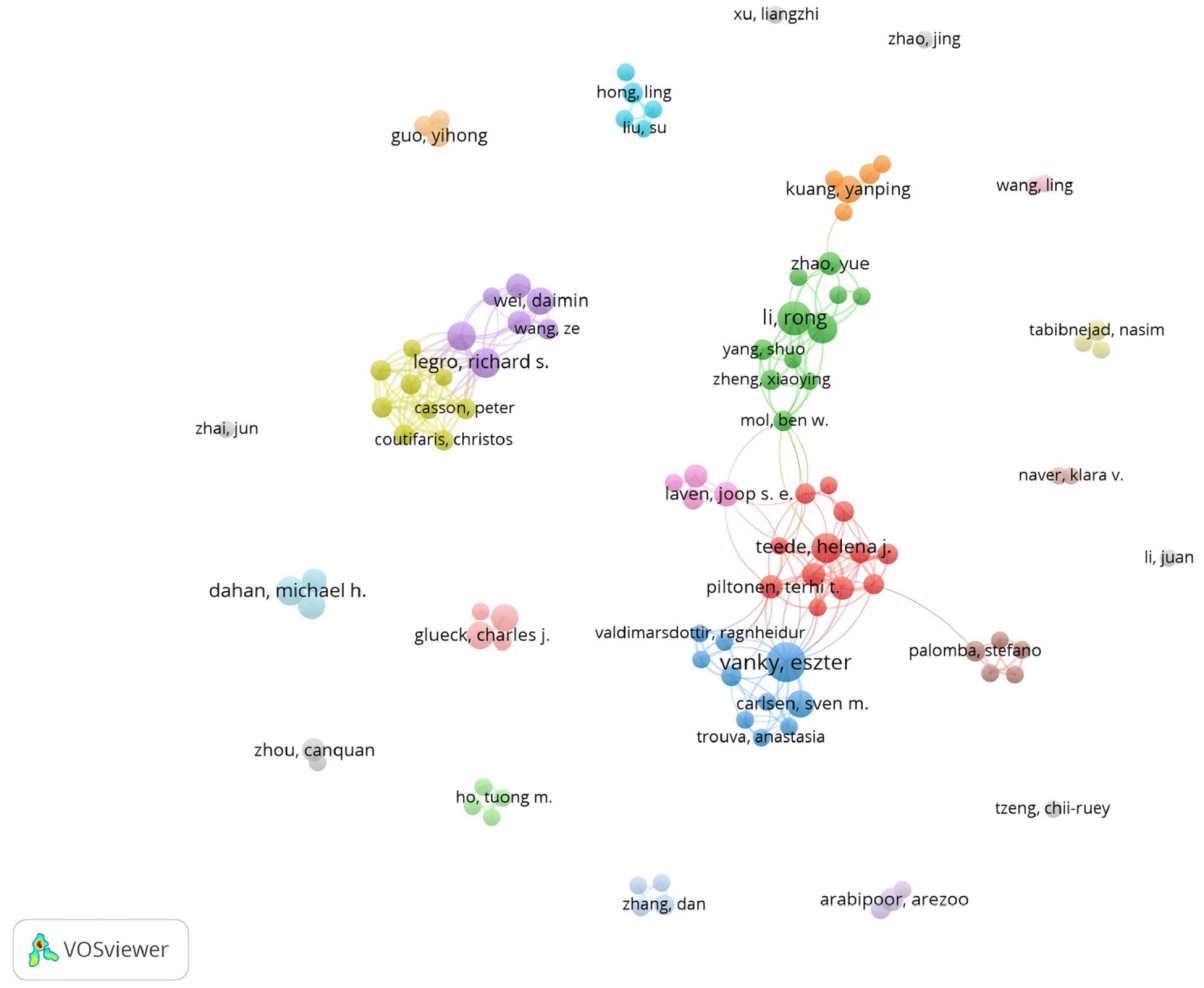
A visualization network of authors with at least 3 published papers. The resulting network clusters 24 distinct groups, where nodes represent authors with larger sizes indicating higher publication counts. Connecting lines demonstrate author relationships, with thicker lines denoting stronger connections. Colors indicate clusters; nodes with identical colors are assigned to the same cluster.

Vanky, Eszter is the most productive scholar, belonging to the Norwegian University of Science and Technology, has been working in this field, published 15 articles. Li Rong secured second place with 11 publications, followed by Legro, Richard S., Teede, Helena J., and Qiao Jie, all contributing 9 papers each. The top 10 authors collectively published at least 7 articles. Notably, Legro, Richard S., demonstrated the highest average citations with 98, highlighting his exceptional research quality. The 103 authors reached the threshold of at least 3 papers per author, and their collaborative network visualization formed 24 clusters (Figure 5).

### 3.5 Journal

**Figure 6:**
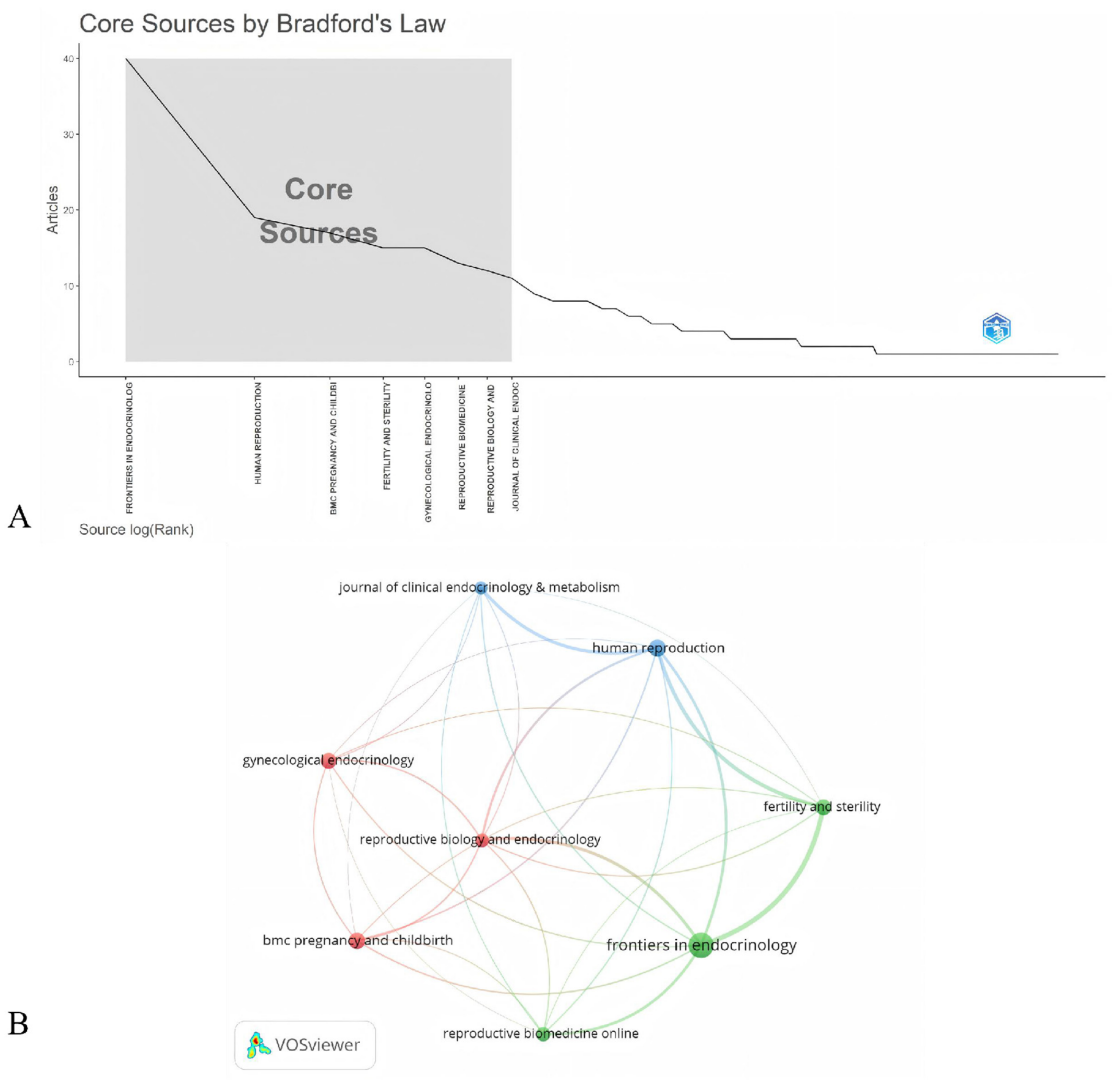
**(A)** Core journal distribution as defined by Bradford's Law. **(B)** The citation network visualization of the top 8 core journals in terms of publication volume formed 3 clusters. Nodes represent journals, with larger nodes indicating higher paper output. Lines denote inter-journal connections, where thicker lines signify stronger correlations. Colors indicate clusters; nodes with identical colors are assigned to the same cluster.

152 academic journals in total have released papers associated with PCOS and pregnancy outcomes. Using Bradford's law, 8 core journals in the field of PCOS and pregnancy outcomes were identified, namely Frontiers in Endocrinology, Human Reproduction, BMC Pregnancy and Childbirth, Fertility and Sterility, and Gynecological Endocrinology (Figure 6A). Table 4 lists the top 8 core journals by number of publications, with Frontiers in Endocrinology leading the list with 40 publications, followed by Human Reproduction, BMC Pregnancy and Childbirth, Fertility and Sterility, and Gynecological Endocrinology. The top 8 journals have impact factors of 1.7–7.0, mostly from the United States and Britain. A citation network (Figure 6B) was constructed based on these 8 core journals, in which Frontiers in Endocrinology (links = 69) and Fertility and Sterility (links = 53) showed the strongest citation relationship.

**Table 4:** The top 8 journals with the most articles.

Rank	Journals	Publications	Impact factor	JCR region	Country
1	Frontiers in Endocrinology	40	4.6	Q1	USA
2	Human Reproduction	19	6.1	Q1	England
3	BMC Pregnancy and Childbirth	17	2.7	Q1	England
4	Fertility and Sterility	15	7.0	Q1	USA
5	Gynecological Endocrinology	15	1.7	Q3	England
6	Reproductive Biomedicine Online	13	3.5	Q1	USA
7	Reproductive Biology and Endocrinology	12	4.7	Q1	England
8	Journal of Clinical Endocrinology & Metabolism	11	5.1	Q1	USA

### 3.6 Keywords and hot frontiers

#### 3.6.1 Co-occurrence analysis of author keywords

**Figure 7:**
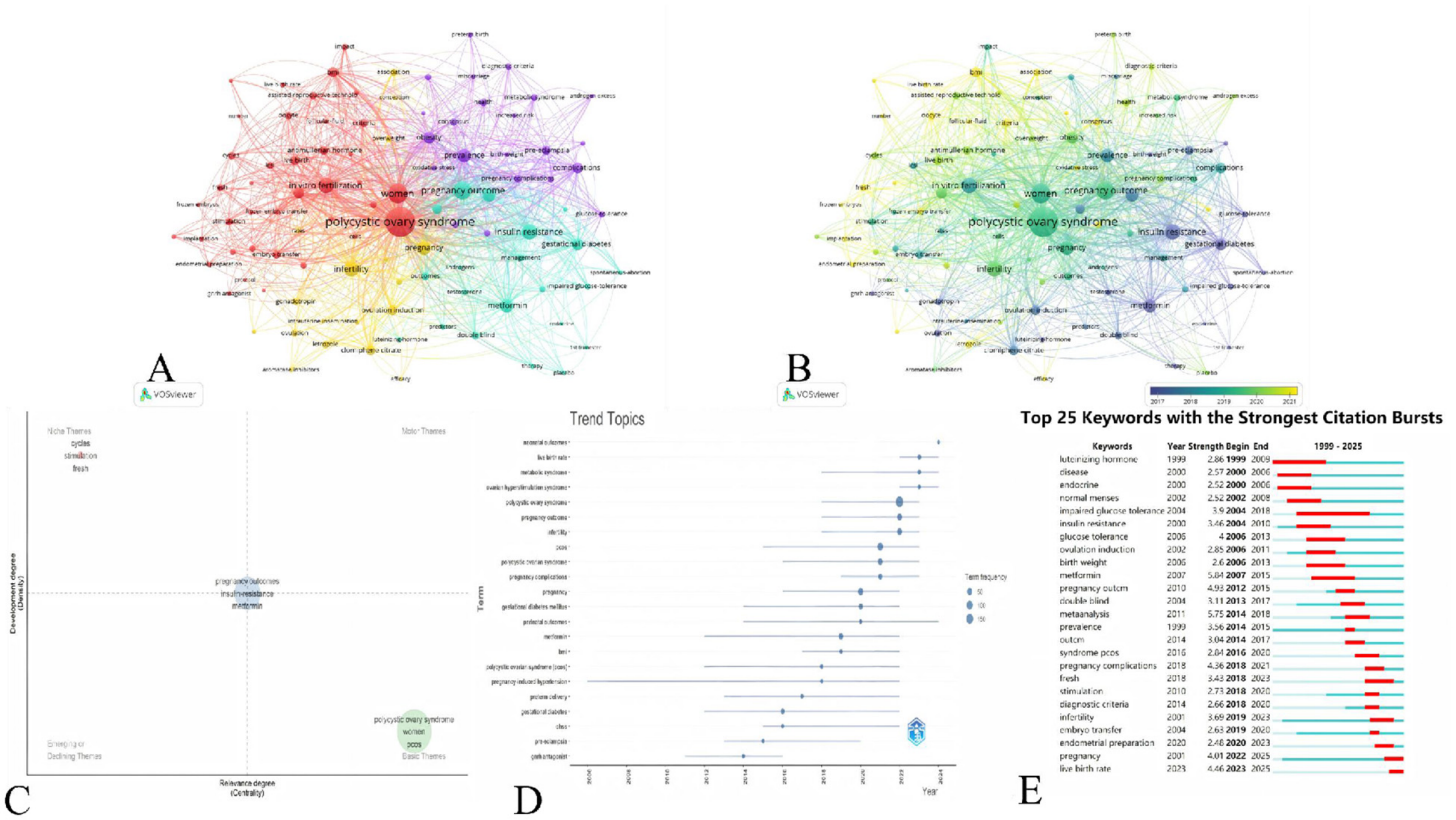
High-frequency keyword map. **(A)** The visualization of the top 100 keywords forms 5 clusters. Nodes symbolize keywords; larger nodes signify higher occurrence rates. Colors indicate clusters; nodes with identical colors are assigned to the same cluster. **(B)** Keywords are distributed by average time of appearance, with blue indicating early and yellow indicating late occurrences. **(C)** The thematic map of the top 250 author keywords with the highest occurrence frequency. **(D)** Trend topics plot for the top 250 author keywords with the highest occurrence frequency. **(E)** Top 25 most cited keywords.

After merging similar keywords, 1,365 keywords were extracted from 410 papers. The top 100 keywords were visualized as shown in Figure 7A, forming 4 clusters. The top 3 most frequent keywords were polycystic ovary syndrome, women, and pregnancy outcomes, with 344, 146, and 126 occurrences, respectively. Based on the keywords provided by the author, we listed the top 5 keywords in each cluster (Table 5). Cluster 1 focuses on ART that support PCOS patients in addressing fertility challenges. Cluster 2 mainly describes pregnancy complications in PCOS patients. Cluster 3 investigates factors influencing pregnancy outcomes in patients with PCOS. Cluster 4 addresses interventions for infertility symptoms in patients with PCOS, including the use of ovulation-inducing agents such as clomiphene citrate (CC). In addition to keyword analysis, we evaluated the recent scholarly attention each field has received. As shown in Figure 7B, the brighter sections predominantly belong to Cluster 1, indicating that ART-related research—particularly frozen embryo transfer—is set to become a core focus in future studies. Furthermore, letrozole is expected to emerge as a key area of interest in upcoming research.

**Table 5:** Keyword clustering.

Cluster	Top 5 keywords
Cluster 1 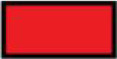	Antimullerian hormone, assisted reproductive technology, BMI, cells, criteria
Cluster 2 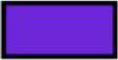	Androgen excess, birth-weight, complications, consensus, diagnostic criteria
Cluster 3 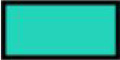	1st trimester, androgens, diagnosis, double-blind, endocrine
Cluster 4 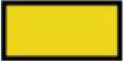	Aromatase inhibitors, association, clomiphene, clomiphene citrate, conception

#### 3.6.2 Hotspot and frontier of PCOS pregnancy outcome research

Figure 7C presents a thematic map that describes the development and correlation of these keywords. Cycles, stimulation, and fresh are located in Niche Themes, representing high density and low centrality. Pregnancy outcomes, insulin resistance, and metformin are situated in the center of the quadrant, which indicates these terms form core topics defined by moderate centrality and density levels. In addition, polycystic ovary syndrome, women, and PCOS are located in the basic theme quadrant, indicating that it represents a highly centrality and low-density basic theme. Therefore, pregnancy outcomes, insulin resistance, and metformin have been identified as hot frontiers in PCOS and pregnancy outcomes research.

#### 3.6.3 Trend topics analysis of author keywords

Through trend theme analysis, Bibliometrix utilizes time series visualization to track the annual evolution of key terms, aiming to identify research trends and emerging frontiers in PCOS and pregnancy outcomes. The parameters specified include a time range from 1999 to 2025, focusing on words that appear at least 50 times and limiting to 3 key terms per year. In Figure 7D, each line represents a topic—its span indicates the length of time, and the circle denotes the peak year of popularity. Circle size reflects topic frequency. The upper left of Figure 7D highlights several new keywords from the past 3 years, indicating that neonatal outcomes, live birth rate, metabolic syndrome, and ovarian hyperstimulation syndrome are emerging frontiers in PCOS and pregnancy outcomes research.

#### 3.6.4 Citation burstness analysis of keywords

We conducted citation burst analysis to pinpoint the top 25 keywords with the greatest intensity. Figure 7E presents their citation bursts, where blue denotes time periods and red indicates burst durations. Citation surges for pregnancy (2022–2025) and live birth rate (2023–2025) continued through 2025, reflecting increased academic interest in these research areas. Metformin demonstrated the strongest citation surge (intensity = 5.84), highlighting its widespread applicability and importance in current research domains. In conclusion, these findings identify pregnancy and live birth rate as emerging frontiers in PCOS and pregnancy outcomes, and metformin as a hot frontier in research.

### 3.7 Analysis of references

#### 3.7.1 Co-citation analysis of references

**Figure 8:**
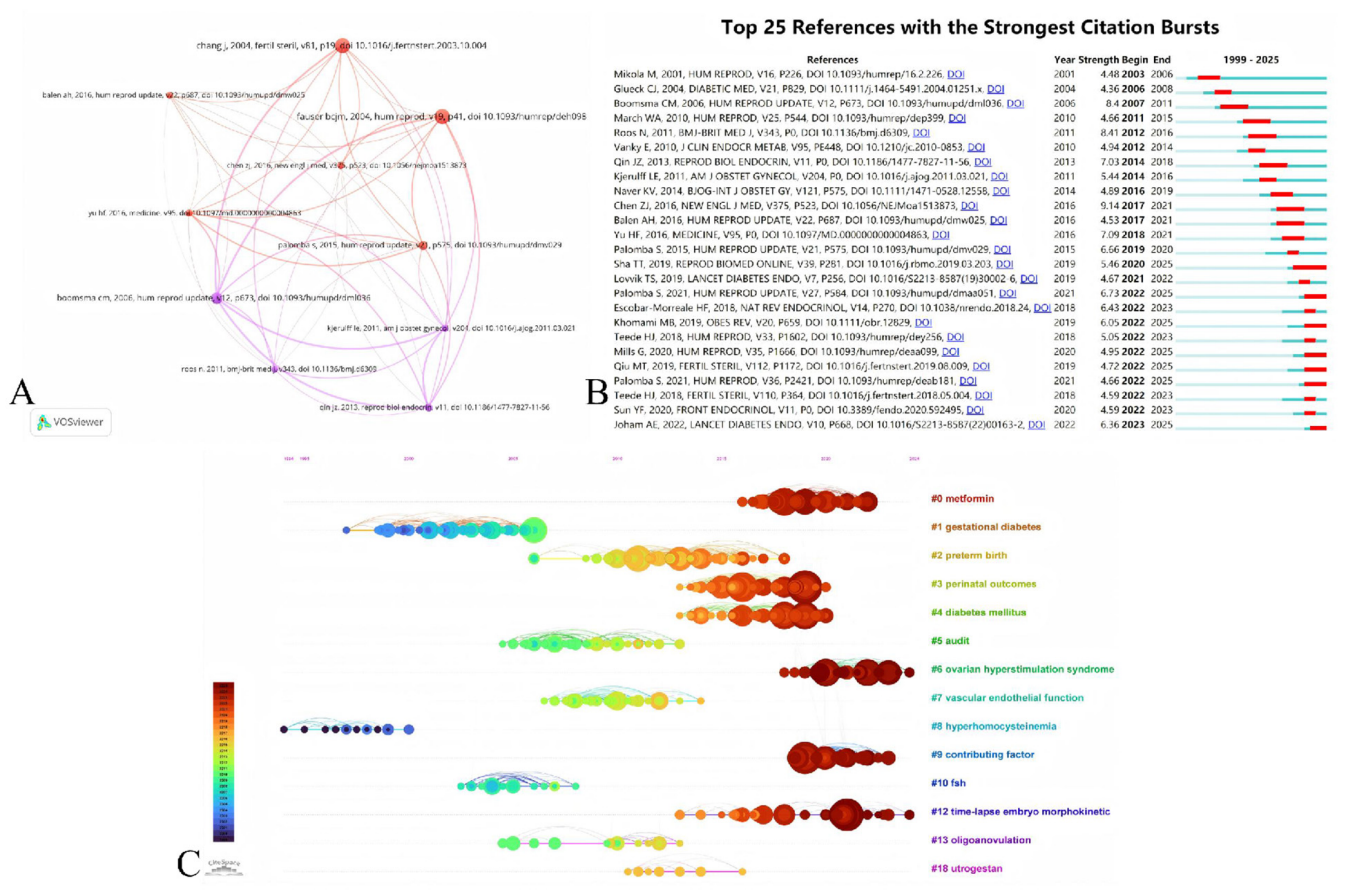
Co-cited literature. **(A)** The network diagram presents a co-citation analysis of the 10 most frequently co-cited references. The size of nodes corresponds to citation frequency, line width denotes the strength of interaction, and different colors differentiate individual clusters. **(B)** The following are the top 25 references with the most significant citation bursts. **(C)** This analysis presents a timeline view of references created by CiteSpace using the log-likelihood ratio (LLR) method.

A total of 11,190 articles were cited by 410 articles, of which the top 3 most cited references were “Rotterdam ESHRE/ASRM-Sponsored PCOS consensus workshop group (89),” “Mirza et al. (25),” and “Boomsama et al. (2).” The top 10 most-cited papers received at least 36 citations, with 2 papers surpassing 120 citations (Table 6). Both papers are identical in content, and both belong to the consensus released by the Rotterdam ESHRE/ASRM-Sponsored PCOS Consensus Workshop Group in 2004. This influential study revised the diagnostic criteria and clarified the long-term health risks associated with PCOS, representing a substantial advancement in PCOS research. Figure 8A shows a co-citation network constructed around the top 10 most cited papers. Within this network, “Boomsama et al. (2)” demonstrated the most robust co-citation relationship with “Qin et al. (90)” (links = 32), “Rotterdam ESHRE/ASRM-Sponsored PCOS consensus workshop group (89)” (links = 32), “Kjerulff et al. (11)” (links = 32), and “Mirza et al. (25)” (links = 30).

**Table 6:** The top 10 publications by citation count.

Rank	Title	Author	Jounal	Year	Impact factor	JCR region	Citations
1	Revised 2003 consensus on diagnostic criteria and long-term health risks related to polycystic ovary syndrome (PCOS)	Fauser, BCJM	Human Reproduction	2004	6.1	Q1	124
2	Revised 2003 consensus on diagnostic criteria and long-term health risks related to polycystic ovary syndrome	Chang, J	Fertility and Sterility	2004	7.0	Q1	123
3	A meta-analysis of pregnancy outcomes in women with polycystic ovary syndrome	Boomsma, CM	Human Reproduction Update	2006	16.1	Q1	84
4	Pregnancy complications in women with polycystic ovary syndrome	Palomba, S	Human Reproduction Update	2015	16.1	Q1	57
5	Obstetric complications in women with polycystic ovary syndrome: a systematic review and meta-analysis	Qin, JZ	Reproductive Biology and Endocrinology	2013	4.7	Q1	46
6	Pregnancy outcomes in women with polycystic ovary syndrome: a meta-analysis	Kjerulff, LE	American Journal of Obstetrics and Gynecology	2011	8.4	Q1	44
7	Fresh vs. Frozen Embryos for Infertility in the Polycystic Ovary Syndrome	Chen, ZJ	New England Journal of Medicine	2016	78.5	Q1	44
8	Association between polycystic ovary syndrome and the risk of pregnancy complications: A PRISMA-compliant systematic review and meta-analysis	Yu, HF	Medicine	2016	1.4	Q4	42
9	Risk of adverse pregnancy outcomes in women with polycystic ovary syndrome: population based cohort study	Roos, N	BMJ-British Medical Journal	2011	42.7	Q1	40
10	The management of anovulatory infertility in women with polycystic ovary syndrome: an analysis of the evidence to support the development of global WHO guidance	Balen, AH	Human Reproduction Update	2016	16.1	Q1	38

#### 3.7.2 Citation burstness analysis of references

After conducting citation explosion analysis on these references, 25 references with the highest explosion intensity were finally identified. Figure 8B describes the citation explosions of these references, with time spans marked in blue and duration of citation explosions shown in red. Over the past 5 years, 7 references, including “Sha et al. (12)” “Palomba et al. (13)” “Khomami et al. (3)” “Mills et al. (14)” “Qiu et al. (15)” and “Joham et al. (16)” have continued to gain prominence. Notably, “Palomba et al. (13)” demonstrated the strongest citation explosion (strength = 6.73), identifying alterations in endometrial function among PCOS women. The study suggests that PCOS clinical features may lead to endometrial expression dysregulation of sex hormone receptors and adrenergic receptors, increasing insulin resistance and impairing glucose transport and utilization in the endometrium. These changes contribute to chronic low-grade inflammation, immune dysfunction, uterine vascular alterations, endometrial gene expression abnormalities, and cellular abnormalities in PCOS patients (13). The citation intensity of “Joham et al. (16)” followed closely (strength = 6.36), which is a comprehensive review of the etiology, diagnosis, treatment, and management of PCOS (16). This was followed by “Khomami et al. (3)” (strength = 6.05), which found that PCOS was linked to a higher risk of pregnancy and delivery complications in the mother, but not to obesity (17). In contrast, “Qiu et al. (15)” (strength = 4.72) showed that obesity led to a progressive decrease in live birth rates and an increase in miscarriage rates among PCOS patients (15). Another article by “Palomba (18)” (strength = 4.66) is an opinion paper that summarizes the collective evidence supporting the hypothesis of reduced fertility potential in PCOS women and speculates that the reduced fertility potential may be caused by changes in oocyte, embryo, and endometrial capacity (18). Additionally, the “Sha et al. (12)” showed a citation explosion (strength = 5.46), providing stronger evidence that women with PCOS undergoing IVF have an increased risk of adverse pregnancy outcomes (12). “Mills et al. (14)” (strength = 4.95) also showed that women with PCOS were twice as likely to develop GDM, 50% more likely to develop hypertensive disorders of pregnancy (GHTN), and 30% more likely to develop preeclampsia (PEC) than women without PCOS (14). Current research examines risk factors contributing to adverse pregnancy outcomes in patients with PCOS.

#### 3.7.3 Timeline view analysis of references

We conducted a timeline view analysis of these references to identify the most common terms in each cluster—these terms served as cluster labels. Figure 8C shows each cluster (numbered #0 to #18), with 19 clusters in total generated via the log-likelihood ratio (LLR) method. A modularity *Q* value of 0.81 indicates a robust clustering effect and close internal links within each cluster. A mean silhouette value of 0.90 indicates that the keywords in each cluster are highly homogeneous and that the differences between clusters are distinct. Clusters related to ovarian hyperstimulation syndrome (#6) and time-lapse embryo morphokinetic (#12) have persisted through 2024. Time-lapse embryo morphokinetic (#12), first introduced in 2013, is a technique that continuously monitors embryonic morphology through frequent imaging. This method provides additional data for morphological evaluation and cell division timing, which determines the outcomes of ART (19, 20). Ovarian hyperstimulation syndrome (# 6) first appeared in 2018. Ovarian hyperstimulation syndrome (OHSS) is a frequent iatrogenic complication resulting from excessive ovulation stimulation. This condition significantly impacts the physical and mental health of infertile women and may increase the risk of perinatal complications in pregnant patients (21, 22). It affects up to 2%−3% of ART participants and can be serious enough to require hospitalization (23). In PCOS women, the risk of OHSS was up to 11 percent (24). Thus, ART-related research has significant implications for the field of PCOS and pregnancy outcomes.

### 3.8 Analysis of clinical experiments

**Figure 9:**
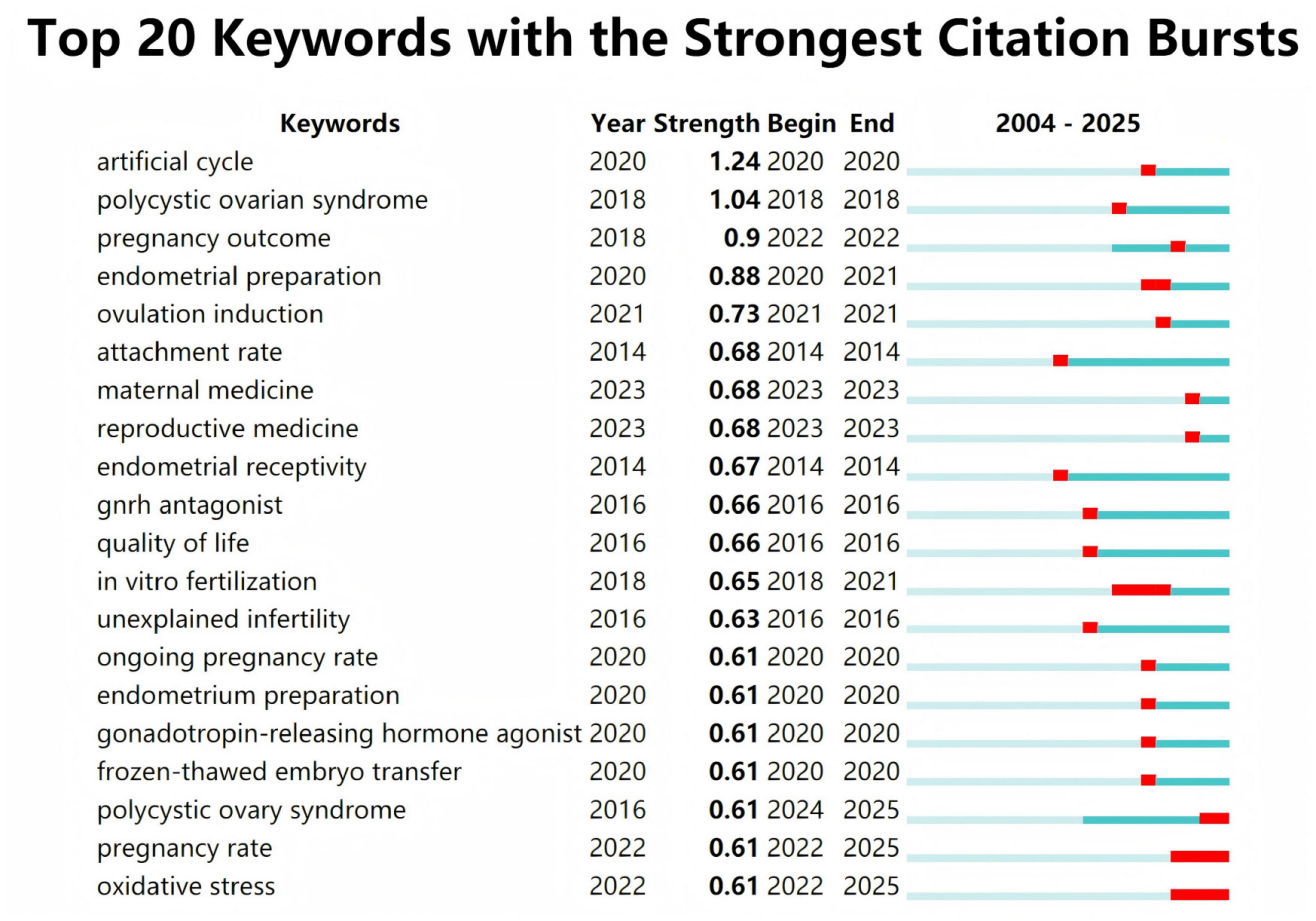
The top 20 most cited keywords for clinical trials in the PubMed database.

A total of 45 clinical trial reports meeting our inclusion criteria were retrieved from the PubMed database. Among these 45 PubMed trials, 80% investigated the impact of different treatment approaches on pregnancy outcomes in PCOS patients, with over half of these trials relating to ART. Through citation burst analysis of keywords, we generated the top 20 most frequently cited keywords (Figure 9). The citation strength for “artificial cycle” was highest (Strength = 1.24), demonstrating ART's significant value and widespread application in PCOS and pregnancy outcomes, thereby validating the trend proposed by WOSCC. The citation counts for “pregnancy rate” and “oxidative stress” persisted through to 2025, indicating the emergence of research into oxidative stress-related mechanisms within this field.

## 4 Discussion

Bibliometrics is a comprehensive framework that combines statistical, mathematical, and linguistic methods to quantitatively assess academic literature in different fields (6). PCOS is the most common endocrine disorder in women and a leading cause of anovulatory infertility. Current research indicates that PCOS presents challenges beyond just conception, with women affected by the condition showing increased risks of adverse pregnancy outcomes, including spontaneous abortion, gestational diabetes, pregnancy-induced hypertension, and preterm birth (25). However, no bibliometric analysis of PCOS and pregnancy outcomes was found in published papers. Therefore, our study represents the first bibliometric analysis examining the relationship between PCOS and pregnancy outcomes, utilizing VOSviewer, CiteSpace, and R software to visualize data from WOSCC and PubMed. The study systematically analyzed 410 papers from WOSCC and 45 clinical trials from PubMed, charting the field's developmental trajectory. It validated the applicability of Price's Law and Bradford's Law in scientometrics and explored high-productivity national institutions, key journals, core authors, keyword clusters, and references within the research domain. Additionally, we conducted a comprehensive analysis of the included papers to identify effective strategies or emerging therapies for treating PCOS-related infertility, aiming to guide clinical diagnosis and treatment.

### 4.1 General discussion

China ranks among the foremost contributors to research on PCOS and pregnancy outcomes, as evidenced by 192 publications. This volume reflects substantial national prioritization and a robust research infrastructure. The United States follows closely with 52 publications. However, a significant disparity exists in the average citation counts between China and the United States, indicating that China still has room for improvement in terms of research depth and influence. Although the Netherlands produces a lower volume of publications, it achieves the highest average citation count, indicating both the quality and international impact of its research output. Moreover, a detailed analysis of inter-institutional collaboration indicates that cooperation among institutions remains limited. The top-ranked institutions are predominantly Chinese. Even at the international level, they rarely join cooperative networks with foreign institutions, choosing instead to collaborate mainly within China. This observation shows that global collaboration in PCOS and pregnancy outcome research remains limited, with networks lacking sufficient breadth. The limited international and inter-institutional collaboration observed in this field may be attributed to several factors: First, language barriers that hinder cross-cultural research communication; Second, differences in research funding systems and grant application procedures across countries; Third, varying PCOS diagnostic criteria and clinical practice norms across regions, which complicate multi-center study design; and the lack of established collaborative frameworks specifically focused on PCOS and pregnancy outcomes. To promote broader collaboration, we recommend establishing international research consortia dedicated to PCOS and pregnancy outcomes, developing standardized multi-center research protocols, encouraging funding agencies to prioritize international collaborative projects, and leveraging existing platforms such as the International PCOS Network to facilitate data sharing and joint research initiatives. Enhanced international cooperation would not only improve the quality and generalizability of research findings but also facilitate the translation of research into clinical practice across diverse healthcare settings.

This analysis highlights leading researchers who have made substantial academic contributions in this field, specifically identifying 2 prominent authors: Vanky, Eszter, and Li, Rong, whose research output stands out quantitatively. Vanky, Eszter's ongoing research and significant publication record demonstrate her professional leadership and emphasize her institution's prominence in the field of PCOS research. Co-authored networks reveal that cross-border collaborative efforts have facilitated global scholarly communication and collaborative initiatives. Furthermore, the authors' scholarly achievements highlight the research strengths and priorities of their regions.

The most highly cited paper among the co-referenced literature is “Revised 2003 consensus on diagnostic criteria and long-term health risks related to polycystic ovary syndrome (PCOS)” (26). This is a consensus published in 2004, developed by the Rotterdam ESHRE/ASRM-Sponsored PCOS consensus workshop group, which is a comprehensive and systematic summary of the diagnosis of PCOS, long-term risk management, and has an important authoritative status. Although this article was published relatively recently, advancements in PCOS research continue to advance. As the authoritative piece in the domain, the citation count of this paper will presumably keep rising with the emergence of new research.

In terms of keywords, we found that the top 100 keywords in terms of citation frequency formed 4 clusters. Cluster 1 is related to ART, and since PCOS is the most common cause of anovulatory infertility, it accounts for 90%−95% of women with anovulatory infertility (27). Therefore, most patients will choose to improve this situation with ART, and with the rapid development of ART, especially *in vitro* fertilization/intracytoplasmic sperm injection and embryo transfer (IVF/ICSI-ET), which is now widely used in PCOS patients (28). IVF/ICSI-ET is effective in resolving ovulatory dysfunction in these patients and can significantly improve infertility outcomes (29). Besides, Cluster 1 also contains antimullerian hormone (AMH). AMH is a promising biomarker in reproductive medicine, with potential applications not only in optimizing ART outcomes and predicting ovarian response but also as a candidate diagnostic criterion for PCOS. A recent comprehensive review examined both the advantages (such as AMH's correlation with ovarian morphology and its relative cycle stability) and the limitations (including assay variability and the need for population-specific cut-off values) of incorporating AMH into PCOS diagnostic criteria. The review underscored the need for standardized assays and further validation before AMH can be routinely integrated into diagnostic frameworks, highlighting both its clinical promise and current challenges in implementation (30). Cluster 2 focuses on pregnancy complications in women with PCOS, which, according to current research, have a higher likelihood of developing GDM and hypertensive disorders (2, 11, 31), which in turn lead to an increased incidence of adverse pregnancy outcomes including fetal growth restriction, preterm labor, and low birth weight, among others (25, 32), as evidenced by a recent meta-analysis involving the offspring of 92,881 women with and without PCOS (3). Cluster 3 primarily explores the etiology and mechanisms underlying pregnancy outcomes in PCOS patients, with adverse pregnancy outcomes in women with PCOS potentially varying according to individual characteristics. Among 3,029 infertile couples in a prospective cohort study, spontaneous pregnancy rates exhibited a linear decrease as body mass index (BMI) increased beyond 29 kg/m^2^. Specifically, this trend indicates a 4% reduction in pregnancy rates for each 1 kg/m^2^ increment in BMI (33). Insulin resistance and hyperinsulinaemia occur in 50%−80% of women with PCOS, and once a patient becomes pregnant, normal pregnancy induces IR and superimposes on pre-pregnancy IR, exacerbating a woman's risk of GDM (2). A study suggests that hyperinsulinaemia, which is present in PCOS, is the main causative mechanism of hypertension in pregnancy, as insulin-sensitive vascular endothelial cells are activated as a result of this hyperinsulinaemic state, which leads to a decrease in the production of prostaglandins, which increases peripheral vascular resistance and leads to an increase in blood pressure (34). About 65 to 80 per cent of PCOS patients have signs or symptoms of HA (35). In contrast, HA may lead to abnormal placental morphology as well as early trophoblastic infiltration and alterations in the placenta, thereby exacerbating the risk of obstetric and perinatal complications (36). Cluster 4 focuses on pharmacological interventions for infertility in PCOS; for patients, pharmacological treatments effective in promoting ovulation, pregnancy, and live births include letrozole and clomiphene citrate, but clomiphene citrate is associated with an increase in the rate of multiple pregnancies, with letrozole and metformin having a relatively minor effect (33). According to recent recommendations, letrozole is recommended as the first line of treatment for ovulation induction in PCOS (37). Clomiphene citrate may also be used in combination with metformin to induce ovulation (38). Additionally, gonadotropins may also be used for ovulation induction. A Meta-analysis examining the efficacy of gonadotropins in inducing ovulation in women with PCOS showed that gonadotropins (FSH) may lead to higher rates of live births and pregnancies than the continued use of CC, without increasing the risk of multiple pregnancies (39).

It should be noted that the quality and level of evidence of the included literature varied considerably. Among the 410 WOSCC publications, 84.15% were original research articles, encompassing study designs ranging from retrospective analyses and cohort studies to randomized controlled trials. The 45 clinical trials retrieved from PubMed were predominantly single-center studies with relatively small sample sizes. Overall, the level of evidence supporting many treatment recommendations in this field remains low to moderate. Future bibliometric analyses in this area could benefit from incorporating quality assessment tools, such as the Jadad scale for clinical trials or the Newcastle-Ottawa Scale for observational studies, to provide a more nuanced evaluation of the evidence base.

For the field of PCOS pregnancy outcome, insulin resistance and metformin are the hot frontiers of research in this field. Emerging frontiers are neonatal outcomes, metabolic syndrome, oxidative stress and ovarian hyperstimulation syndrome, and future hotspots may be letrozole and frozen embryo transfer. It can be seen that the focus of research on pregnancy outcomes in PCOS can be divided into 2 main categories: firstly, influencing factors and mechanisms; and secondly, diagnosis and treatment.

### 4.2 Treatment of PCOS infertility

#### 4.2.1 Letrozole and clomiphene citrate

According to recent guidelines (37), the first-line therapeutic agent for infertility in patients with PCOS is letrozole. By combing through 410 publications, we found that most scholars believe that oral letrozole alone is not as effective as letrozole combination therapy. A study found that oral letrozole combined with low-dose injections of human menopausal gonadotropin (HMG) significantly increased the clinical pregnancy rate of patients, and the number of ovulatory cycles was significantly higher than that of the letrozole-only group (40). Similar conclusions were reached in a study by Dai et al. (41), who found that the letrozole combined with HMG treatment group had a significantly higher rate of ovulation (90.8%) and live births (23.0%) than that of the letrozole group (70.1% ovulation rate; 10.3% live births). However, both letrozole alone and the combination regimen used for endometrial preparation for frozen embryo transfer (FET) showed similar pregnancy outcomes to those of artificial cycle endometrial preparation (AC-FET), even though the incidence of gestational diabetes mellitus was significantly higher in the letrozole alone group (42, 43). In addition, letrozole in combination with exercise is also excellent, and studies have shown that the clinical pregnancy rate was significantly higher in the letrozole in combination with exercise group (69.8%) compared to the letrozole alone group (31.4%) (44). As for the optimal starting time for oral letrozole, it is currently common in clinical practice to begin oral administration on days 3–5 of the menstrual cycle. However, oral administration on day 5 is more effective, and patients are more likely to ovulate, conceive, and become pregnant (45). Clomiphene Citrate (CC) is also an effective ovulation-promoting drug, although most studies have demonstrated that letrozole is superior to CC in terms of ovulation, pregnancy, and live birth rates (46, 47). However, a simulated hypothetical trial (48) comparing letrozole with CC for ovulation induction in all women found that in women with unexplained infertility, the 2 drug treatments resulted in comparable rates of live births; in women with PCOS, the pregnancy rates in the letrozole group vs. the CC group were 45 per cent and 39 per cent, respectively, and the live birth rates were 34 per cent and 32 per cent, respectively. Therefore, we recommend letrozole as the first-line drug for women with PCOS infertility, and the combination therapy is more effective than oral alone, but the combination therapy with the best efficacy has not been found yet, and more scholars need to carry out in-depth research, which may become a hotspot for future research.

#### 4.2.2 Metformin (MTF)

MTF, a hypoglycaemic agent, has emerged as a potential drug for the treatment of infertility in women with PCOS due to its significant efficacy in promoting ovulation, improving insulin sensitivity, and facilitating clinical pregnancy in women with PCOS, with a low multiple pregnancy rate (16, 49). Research on MTF has been ongoing, and the question of whether MTF can improve pregnancy outcomes in women with PCOS remains controversial. However, after reading all the literature included, we found that most of the findings demonstrated that MTF significantly reduced the rates of OHSS, miscarriage, and pregnancy complications, but did not improve the pregnancy and live birth rates (50–57). Jacob et al. (58) concluded that short-term use of MTF in women with PCOS receiving cycles of GnRH antagonist therapy did not reduce the incidence of OHSS and even reduced pregnancy rates, but they considered this result to be fortuitous. Some studies have shown that MTF treatment enhances pregnancy and live birth rates compared to placebo, but the quality of the evidence is low, so this conclusion still needs to be validated by more robust trials (59–61). The results of a large randomized double-blind controlled trial demonstrated that MTF treatment of women with PCOS may reduce their risk of late miscarriage and preterm delivery and does not prevent gestational diabetes (62), but MTF has better efficacy in patients with higher BMI (49). Whether MTF can be recommended for treating infertility in women with PCOS remains to be verified by additional studies. Currently, evidence suggests MTF may be more suitable for PCOS women with a higher BMI or those complicated by OHSS, and it is not advisable to use it as a first-line treatment.

#### 4.2.3 Gonadotropin

Gonadotropins are used as second-line therapy for ovulation induction in women with PCOS (39), but as they lead to higher rates of multiple pregnancies and OHSS (63), they are usually taken as injections after letrozole or other medications, which can reduce complications and maintain high pregnancy rates (40, 41). This approach was first proposed by Yun (64), Kistner (91). Since then, researchers have investigated the efficacy of combining various drugs with different doses and types of gonadotropins in ovulation induction therapy. In recent years, it has been found that the use of LE in combination with HMG injection protocols in frozen embryo transfer (FET) and intrauterine insemination (IUI) has yielded favorable results (42, 65). So which gonadotropin is more efficacious? A study comparing the efficacy of HMG and recombinant follicle stimulating hormone (rFSH) combined with CC for ovulation promotion in patients with PCOS before undergoing IUI showed that there was no significant difference between the HMG+CC and rFSH+CC groups in terms of the clinical pregnancy rate, the rate of sustained pregnancies, the rate of live births, the rate of miscarriages, and the incidence of OHSS, whereas in the rFSH group the duration of treatment was shorter than that of the HMG group, and the overall dosage was also lower and the mean follicle diameter and endometrial thickness were also higher. Therefore, they concluded that rFSH is more efficacious than HMG in IUI cycles (66). However, a recent Meta-analysis comparing pregnancy outcomes between different gonadotropins found that rFSH and purified FSH (uFSH) had similar outcomes in terms of live birth rate and pregnancy rate. And it is uncertain whether either HMG or uFSH can improve their live birth and pregnancy rates, as the quality of evidence from all the included studies was very low (39). Therefore, large-scale, multicenter randomized controlled trials (RCTs) are still needed to compare the efficacy of different gonadotropin therapies.

#### 4.2.4 ART

For PCOS patients, ART stands as the ultimate treatment for infertility issues. Previously, Chen's team has demonstrated through a large multicentre randomized clinical trial that Frozen Embryo Transfer (FET) significantly improves the live birth rate and reduces the risk of OHSS compared to fresh embryo transfer in women with PCOS infertility (67), and therefore, FET is more recommended for women with PCOS. Recently, Chen's team's new research showed that among women with poor IVF prognosis (excluding those with PCOS) who underwent fresh embryo transfer or FET, the fresh embryo transfer group exhibited higher live birth rates, clinical pregnancy rates, and cumulative live birth rates compared with the FET group (68). This demonstrates that fresh embryo transfer is superior to FET for non-PCOS women with poor IVF prognosis. Therefore, in the future, perhaps comparing fresh embryo transfer and FET pregnancy outcomes in women with PCOS who have a poor prognosis for IVF is a new direction for research. In recent years, the majority of scholars have investigated the impact of gonadotropin-releasing hormone agonist (GnRH-a) pretreatment on pregnancy outcomes in PCOS patients undergoing FET. A retrospective analysis revealed that GnRH-a pretreatment significantly reduced miscarriage rates, yet demonstrated no substantial difference in pregnancy or live birth rates compared to outcomes without pretreatment (69). Luo et al.'s (70) RCT included 343 patients, and they reached similar conclusions. This was demonstrated in a subsequent Meta-analysis (71), where GnRH-a pretreatment did not offer any advantage to women with PCOS undergoing FET and did not significantly improve pregnancy outcomes. Whereas GnRH-a pretreatment significantly enhanced the clinical pregnancy rate in patients with recurrent implantation failure (RIF), it was not statistically significant in patients with PCOS combined with RIF, although the pregnancy rate was higher than that in the control group (72). However, a retrospective study that included 1,771 came to a very different conclusion when they concluded that pretreatment with long-acting GnRH-a in the early follicular phase improves the rate of live births in FET cycles. In particular, age < 40 years, primary infertility, PCOS, and menstrual irregularities are valid indications for endometrial preparation with GnRH-a pretreatment in FET cycles (73). We believe that the reason for this controversy lies in the differing durations of GnRH-a administration and patient populations included across studies. Large-scale, multicentre, double-blind randomized controlled trials are still required to validate the impact of this intervention on pregnancy outcomes in women with PCOS. As GnRH agonists cause problems such as hypo-oestrogenic symptoms, multiple pregnancies, and a high incidence of OHSS (74), a milder stimulation regimen has been introduced—namely, GnRH antagonists (GnRH-ant), which decreases the rate of multiple pregnancies and the OHSS incidence and has a more rapid onset of action (75). Most studies have found similar pregnancy outcomes with the GnRH-ant regimen compared to the GnRH-a regimen in women with PCOS treated with IVF (76, 77). However, the findings of Hosseini et al. (78) showed significantly higher numbers of oocytes retrieved and significantly lower incidence of OHSS in the antagonist group in terms of chemical and clinical pregnancy rates. In conclusion, GnRH-ant can be used as an alternative to GnRH-a in assisted reproduction cycles in PCOS patients and is more effective in normal-weight women (79).

#### 4.2.5 Traditional Chinese medicine (TCM)

TCM treatment for PCOS infertility is an emerging therapy for international purposes, but has become the mainstay in China, often in combination with letrozole or CC. Acupuncture and Moxibustion has been shown to significantly improve ovulation promotion and pregnancy outcomes in women with PCOS. Supporting this, a Mesh Meta-analysis (80) demonstrated that when comparing interventions for pregnancy outcome improvement, the acupuncture & moxibustion plus clomiphene combination was superior to both monotherapy with acupuncture and moxibustion and monotherapy with clomiphene, and monotherapy with acupuncture and moxibustion was superior to monotherapy with clomiphene. Acupuncture and Moxibustion combined with clomiphene group was the most advantageous in improving endometrial thickness, and electroacupuncture combined with clomiphene was the most effective in improving ovulation rate. In addition, the incidence of adverse effects was greatly reduced in the acupuncture and moxibustion combined with clomiphene group compared to the clomiphene group. Another network meta-analysis likewise did a similar study, where they found that the treatment combination of CC plus warm acupuncture (WA) and letrozole plus manual acupuncture (MA) achieved the highest probability of achieving the best pregnancy outcome (81). So, is there a correlation between the dose of acupuncture and pregnancy outcomes? Li et al. (82) provided the answer: the higher the acupuncture dosage, the more favorable the pregnancy outcomes for PCOS patients. In addition, 2 Meta-analyses have investigated the effects of Chinese herbal medicine on the pregnancy outcomes of PCOS patients undergoing IVF-ET, and found that Chinese herbal medicine treatment was effective in improving the IVF-ET pregnancy outcomes of patients with PCOS, and significantly increased the clinical pregnancy rates of the patients (83, 84). However, Zhang et al. (85) found that Chinese herbs alone did not significantly improve pregnancy or ovulation rates, while Chinese herbs plus clomiphene boosted pregnancy rates. These results are less convincing, though, as the meta-analysis only included 4 RCTs and the studies were of low quality. Chinese medicine monomers quercetin and berberine also improve pregnancy outcomes in women with PCOS, significantly increasing pregnancy rates and live birth rates while reducing the incidence of adverse reactions (86, 87). TCM treatments encompass herbal medicine, acupuncture, moxibustion, acupoint application, and acupoint catgut embedding, among others. However, within these publications, we identified only herbal medicine, acupuncture, and monomers of Chinese traditional herbs. Moreover, the majority of these studies were published within the last 5 years. This indicates that TCM holds significant untapped potential in researching pregnancy outcomes for women with PCOS and may emerge as a future research focus in this field.

### 4.3 Recommendation

For clinicians, the study provides the following recommendations: Letrozole can be given priority as the first-line ovulation induction drug for infertile patients with PCOS, and metformin can be combined for those with high BMI or at risk of OHSS. Frozen embryo transfer is recommended in ART to reduce the risk of OHSS. TCM, such as acupuncture and Chinese herbal medicine, can be used as an adjuvant treatment to improve ovulation rate and clinical pregnancy rate when combined with Western medicine. Pregnancy management of PCOS patients needs to focus on monitoring insulin resistance and metabolic syndrome, and timely intervention to reduce the risk of complications such as gestational diabetes mellitus and preeclampsia.

For researchers, we recommend prioritizing the following areas: In the future, large-scale, multicentre, randomized controlled trials could compare different letrozole combination regimens. It is also important to investigate the mechanisms linking metabolic syndrome, insulin resistance, or oxidative stress to adverse pregnancy outcomes. Researchers should conduct comparative studies of fresh vs. frozen embryo transfer in PCOS patients with poor IVF prognosis. Standardizing TCM adjuvant protocols is recommended to improve the evidence-based level of PCOS pregnancy management.

### 4.4 Limitations

This study has several limitations. First, only English-language publications were included in this analysis, which may introduce language bias and exclude potentially relevant studies published in other languages. This is particularly noteworthy given that China is the leading contributor in this field, and a substantial body of Chinese-language research on PCOS and pregnancy outcomes likely exists in domestic databases such as CNKI and Wanfang. Additionally, animal studies and genetic studies were excluded, which may lead to an incomplete research perspective. Second, obvious heterogeneity existed due to diverse clinical practice norms, inconsistent PCOS diagnostic criteria, variable definitions of pregnancy outcome indicators, and different medicine/ART regimens across regions. Third, publication bias may exist due to the omission of gray literature (such as conference abstracts and dissertations) and a higher publication probability for positive results. Fourth, the predominantly small-sample, single-center nature of included clinical trials, resulting in an overall low level of evidence for the conclusions. Fifth, bibliometric analysis inherently relies on quantitative metrics (publication counts, citation numbers) that may not fully reflect research quality, clinical significance, or real-world impact of individual studies. Finally, the quality and integrity of contributions from different regions and institutions were not individually assessed, which may affect the representativeness of our findings. We encourage future studies to explore this dimension. A more comprehensive search strategy should be implemented, integrating analysis results from multiple databases. Additionally, it's important to include high-quality studies to enhance the reliability and generalizability of the research conclusions.

## 5 Conclusion

This study represents the first bibliometric analysis focused on PCOS and pregnancy outcomes, systematically analyzing 410 publications from the WOSCC database and 45 clinical trials from PubMed. China and the United States are the leading contributors to this field, though international collaboration remains limited and should be strengthened. Insulin resistance and metformin are current research hotspots, while neonatal outcomes, metabolic syndrome, oxidative stress and ovarian hyperstimulation syndrome represent emerging frontiers. Among treatment strategies, letrozole is recommended as the first-line ovulation induction agent, frozen embryo transfer is preferred in ART to reduce OHSS risk, and TCM shows promising potential as adjunctive therapy. Future research should prioritize large-scale, multi-center RCTs to strengthen the evidence base, and enhanced international collaboration is essential to advance the field. Clinicians can leverage these findings to guide evidence-based management of PCOS patients seeking pregnancy, while researchers can use the identified knowledge gaps to direct future investigations.

## Data Availability

The raw data supporting the conclusions of this article will be made available by the authors, without undue reservation.
