# Supplementary material for: Investigation of trends, hot spots and effective therapies for pregnancy outcomes in polycystic ovary syndrome: a bibliometric analysis
Source: Front Med (Lausanne). 2026 May 7;13:1756551. doi: 10.3389/fmed.2026.1756551 (PMC13220317; doi:10.3389/fmed.2026.1756551)
Supplement: Supplementary file 1 [file Table_1.docx]

Supplementary Table 1 Detailed search strategy

| **Web of Science database search strategy**  [TS= (“Polycystic Ovary Syndrome” OR “Syndrome, Polycystic Ovary” OR “Ovary Syndrome, Polycystic” OR “Stein Leventhal Syndrome” OR “Stein-Leventhal Syndrome” OR “Syndrome, Stein-Leventhal” OR “Ovarian Degeneration, Sclerocystic” OR “Sclerocystic Ovarian Degeneration” OR “Sclerocystic Ovary Syndrome” OR “Ovarian Syndrome, Polycystic” OR “Polycystic Ovarian Syndrome” OR “Sclerocystic Ovaries” OR “Sclerocystic Ovary” OR “Ovary, Sclerocystic”) AND TS=(“Pregnancy outcome” OR “Outcome, Pregnancy” OR “Outcomes, Pregnancy” OR “Pregnancy Outcomes”)] | **PubMed database search strategy**  [("Polycystic Ovary Syndrome"[Title/Abstract] OR "Syndrome, Polycystic Ovary"[Title/Abstract] OR "Ovary Syndrome, Polycystic"[Title/Abstract] OR "Stein Leventhal Syndrome"[Title/Abstract] OR "Stein-Leventhal Syndrome"[Title/Abstract] OR "Syndrome, Stein-Leventhal"[Title/Abstract] OR "Ovarian Degeneration, Sclerocystic"[Title/Abstract] OR "Sclerocystic Ovarian Degeneration"[Title/Abstract] OR "Sclerocystic Ovary Syndrome"[Title/Abstract] OR "Ovarian Syndrome, Polycystic"[Title/Abstract] OR "Polycystic Ovarian Syndrome"[Title/Abstract] OR "Sclerocystic Ovaries"[Title/Abstract] OR "Sclerocystic Ovary"[Title/Abstract] OR "Ovary, Sclerocystic"[Title/Abstract]) AND ("Pregnancy outcome"[Title/Abstract] OR "Outcome, Pregnancy"[Title/Abstract] OR "Outcomes, Pregnancy"[Title/Abstract] OR "Pregnancy Outcomes"[Title/Abstract]) Filters: Clinical Trial, English, from 1000/1/1 - 2025/5/10] |
| --- | --- |
